# NUrse-led COntinuum of care for people with Diabetes and prediabetes (NUCOD) in Nepal: study protocol for a cluster randomized controlled trial

**DOI:** 10.1186/s13063-020-04372-5

**Published:** 2020-05-29

**Authors:** Dong ( Roman) Xu, Rubee Dev, Abha Shrestha, Lingling Zhang, Archana Shrestha, Pushpanjali Shakya, James P. Hughes, Prabin Raj Shakya, Jinghua Li, Jing Liao, Biraj Man Karmacharya

**Affiliations:** 1 Sun Yat-sen University Global Health Institute (SGHI), Sun Yat-sen University, Guangzhou, China; 2 Department of Community Medicine, Kathmandu University School of Medical Sciences, Dhulikhel, Nepal; 3 College of Nursing and Health Sciences, University of Massachusetts, Boston, MA USA; 4 Department of Epidemiology, Harvard TH Chan School of Public Health, Boston, MA USA; 5 Department of Biostatistics, University of Washington, Seattle, WA USA; 6 College of Dentistry, Biomedical Knowledge Engineer Lab, Seoul National University, Seoul, Korea; 7 Department of Community Programs, Kathmandu University School of Medical Sciences, Dhulikhel Hospital, Kavre, Nepal

**Keywords:** Continuum of care, Diabetes, Prediabetes, Randomized controlled trial, Implementation

## Abstract

**Background:**

The purpose of this study will be to improve diabetes prevention, access to care and advocacy through a novel cost-effective nurse-led continuum of care approach that incorporates diabetes prevention, awareness, screening and management for low-income settings, and furthermore utilizes the endeavor to advocate for establishing a standard diabetes program in Nepal.

**Methods:**

We will conduct a two-arm, parallel group, stratified cluster randomized controlled trial of the NUrse-led COntinuum of care for people with Diabetes (*N*_1_ = 200) and prediabetes (*N*_2_ = 1036) (NUCOD) program, with primary care centers (9 outreach centers and 17 government health posts) as a unit of randomization. The NUCOD program will be delivered through the trained diabetes nurses in the community to the intervention group and the outcomes will be compared with the usual treatment group at 6 and 12 months of the intervention. The primary outcome will be the change in glycated hemoglobin (HbA1c) level among diabetes individuals and progression to type 2 diabetes among prediabetes individuals, and implementation outcomes measured using the RE-AIM (reach, effectiveness, adoption, implementation and maintenance) framework. Outcomes will be analyzed on an intention-to-treat basis.

**Discussion:**

The results of this trial will provide information about the effectiveness of the NUCOD program in improving clinical outcomes for diabetes and prediabetes individuals, and implementation outcomes for the organization. The continuum of care model can be used for the prevention and management of diabetes and other noncommunicable diseases within and beyond Nepal with similar context.

**Trial registration:**

ClinicalTrials.gov, NCT04131257. Registered on 18 October 2019.

## Background

Prevalence of type 2 diabetes (hereafter, diabetes) has been rising like a silent epidemic in Nepal, similar to other developing countries. A systematic review and meta-analysis reported a pooled prevalence of type 2 diabetes of 8.4% (95% CI: 6.2–10.5%) [1]. It is estimated that between 1990 and 2010 the burden of diabetes increased by about 89% in Nepal [2].

The most recently published Disease Control Priority-3 (DCP-3) systematically reviewed available interventions for diabetes in resource-limited settings, and strongly recommended the following interventions based on their cost-effectiveness and feasibility: targeted (two-step) screening for both prediabetes and diabetes; blood pressure control among people with diabetes; lifestyle interventions to prevent diabetes among high-risk individuals; and good glycemic control along with smoking cessation and foot care. Those interventions, ranging from case detection to prevention of diabetes among those at high risk, to diabetes management and to screening for preventing diabetes complications, have been extensively tested for efficacy and cost-effectiveness. However, the DCP-3 pointed out two knowledge gaps: little evidence for the testing of the effectiveness of those interventions in low-income settings; and a lack of implementation-oriented research for adopting and scaling up those interventions. This is compounded by the fact that a chronic disease like diabetes needs a structured, well-concerted comprehensive approach comprising continuum of care prevention activities, timely screening, standard clinical management and self-management skills for lifestyle modification rather than segregated interventions. Nurse care coordinators have been found to improve patient and health service outcomes, particularly when they frequently interacted with the patients, conducted follow-up with monitoring and education of the disease and behavioral changes, and were involved in transition care [3–7]. However, they often focused on specific components rather than the full spectrum of the continuum of care for the patient. Also, studies are rarely conducted in low-income countries.

The Dhulikhel Hospital (DH) system is a community-oriented, nongovernmental health system with an extensive network of 21 rural community-based outreach centers. The DH system provides a well-suited setting for innovating a continuum of care for people with diabetes. In this trial, we aim to develop and implement a “continuum of care” program with trained nurses as the nucleus. The nurses, along with the community health worker, will coordinate and implement a composite of the DCP-3-recommended individual intervention components for the low-income setting: leading community awareness campaigns; organizing diabetes screening events; linking diabetic patients to comprehensive clinical care (which will have been structured based on standard guidelines); and ensuring treatment adherence and self-management through regular community-based group counseling programs. The primary objective of this study is to examine the effectiveness of this comprehensive intervention in promoting the health of the people with diabetes and prediabetes, and the secondary objective is to examine whether the nurse coordination will promote the implementation of the evidence-based, DCP-3-recommended interventions. We hypothesize that the NUrse-led COntinuum of care for people with Diabetes and prediabetes (NUCOD) program will improve both clinical outcomes as well as implementation outcomes, compared with diabetes usual care in low-income settings.

## Methods

### Design

**Fig. 1:**
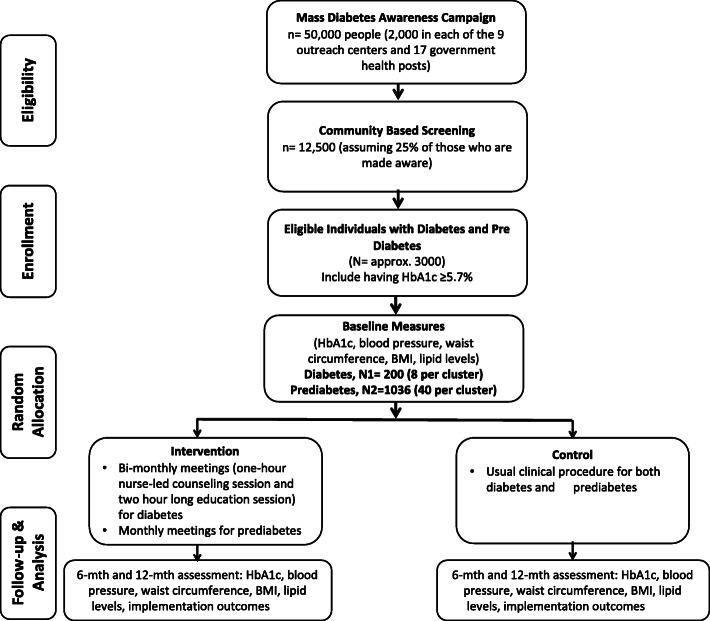
Summary of the trial design for the NUCOD program. BMI body mass index, HbA1c hemoglobin A1c (aka glycated hemoglobin), NUCOD NUrse-led COntinuum of care for people with Diabetes and prediabetes

This study will be a two-armed, parallel group, stratified cluster randomized controlled trial (RCT) of the NUCOD program (Fig. 1), with the health center as a unit of randomization. We will use a computer-generated list of random numbers to randomize health centers stratified by Kathmandu University (KU) outreach centers and government health posts, whereby all patients from the same health center will be allocated to the same group. The study will be planned and implemented in concordance with the Consolidated Standards of Reporting Trials (CONSORT) cluster trial extension statement [8] and the Standards for Reporting Implementation Studies (StaRI) statement [9]. We plan to use the cluster design to reduce between-group contamination and spillover effects of the intervention as well as to align the design with the natural project implementation unit based on the cluster.

### Setting

The study will be conducted in 26 clusters: 9 outreach centers (Bahunepati, Manekharka, Hindi Health, Baluwa, Bolde Phediche, Dapcha, Kartike Deurali, Salambu and Dhunkharka Health Centers) of Dhulikhel Hospital, Kathmandu University Hospital and 17 government health posts located in the adjoining catchment areas purposively selected from the Kavrepalanchowk and Sindhupalchowk districts. The primary stratification variable, as well as the unit (cluster) of randomization and implementation, will be health clinics (outreach centers and health posts (HPs)). There will be 13 clusters per treatment arm (1:1 allocation ratio). Outreach centers are the small medical health facilities that offer basic and emergency care services in remote areas of Nepal. These centers are run by Kathmandu University Hospital. Similarly, health posts are government-run facilities that provide basic health care and preventive medication in remote rural areas of Nepal.

**Fig. 2:**
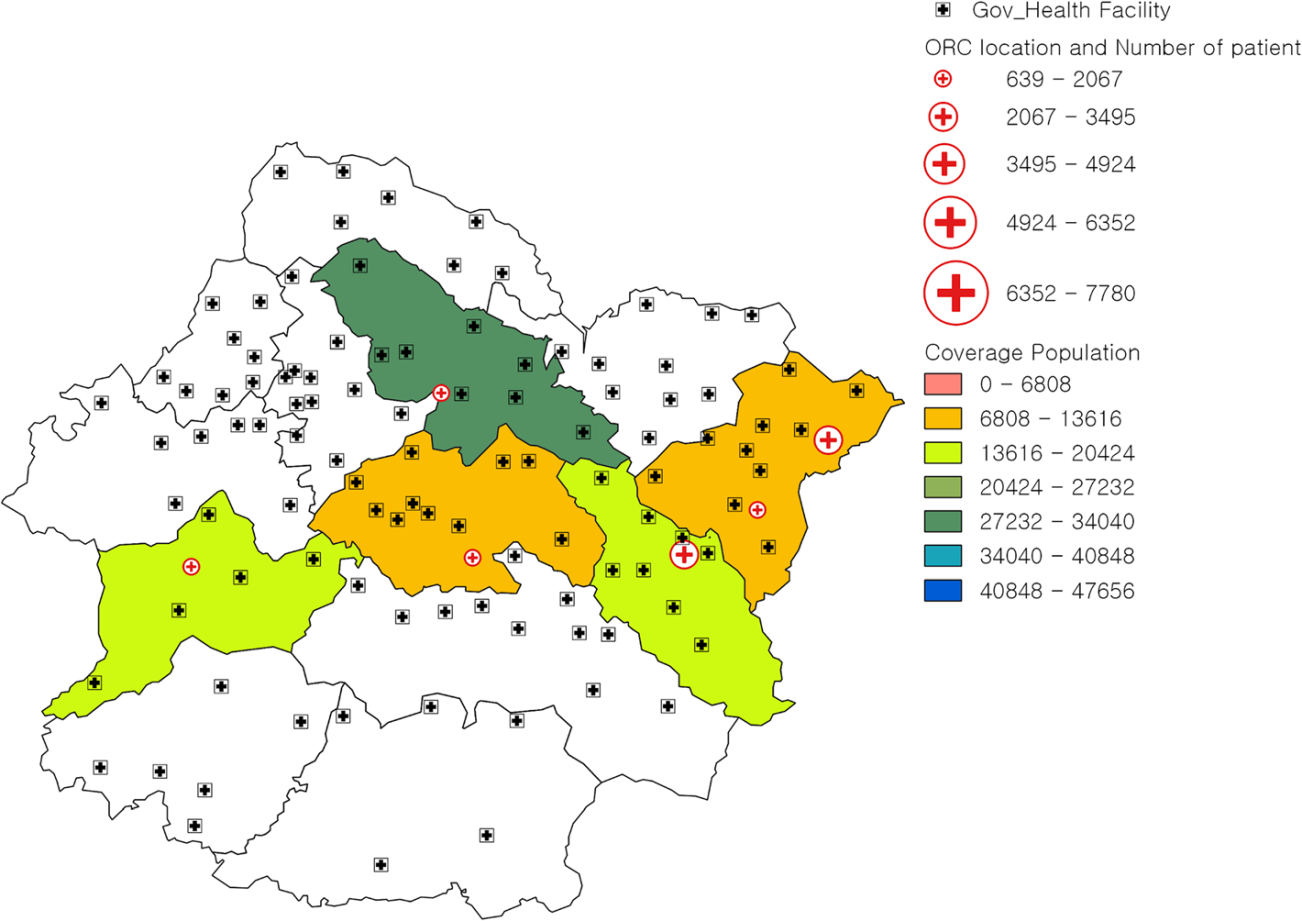
Map of Kavre District with outreach centers of Dhulikhel Hospital and government facilities. ORC outreach center

**Fig. 3:**
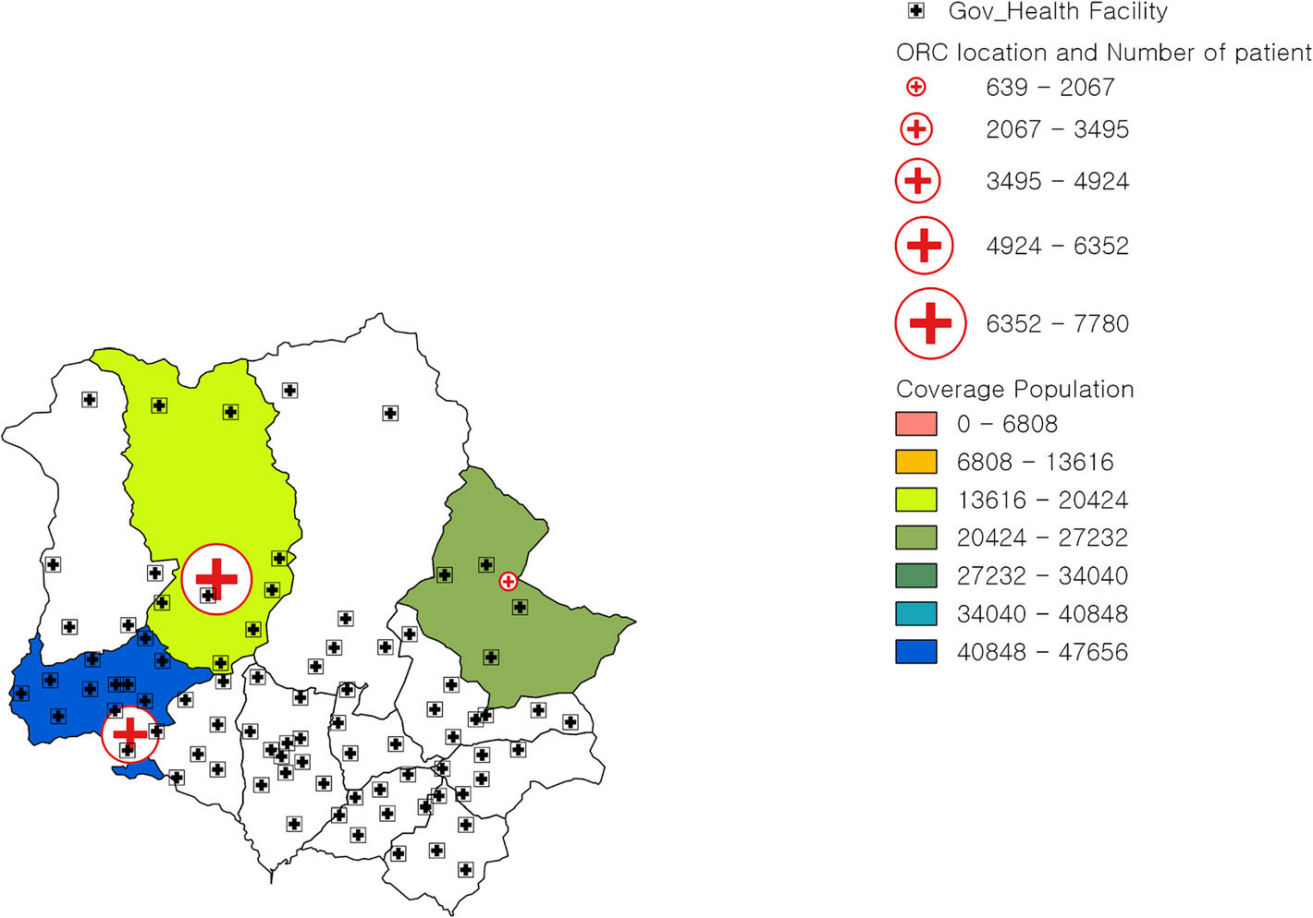
Map of Sindhupalchowk District with outreach centers of Dhulikhel Hospital and government facilities. ORC outreach center

Kavrepalanchowk district located in Province 3 of Nepal consists of 13 municipalities: 6 urban and 7 rural municipalities with a total of 137 wards. The headquarter of this district is Dhulikhel municipality. A total population of 381,937 resides in this district; 62.51% live in the urban municipalities and 37.49% live in the rural municipalities. There is one 15-bed district hospital, 4 primary health care (PHC) centers and 86 HPs in the district [10]. Sindhupalchowk district, also located in Province 3 of Nepal, consists of 12 municipalities: 3 urban and 9 rural municipalities with a total of 103 wards. The headquarter of Sindhupalchowk district is Chautara. A total population of 287,798 resides in this district; 41.42% live in the urban municipalities and 58.58% live in the rural municipalities. There is one 15-bed district hospital, 3 PHC centers and 75 HPs in the district [10]. The maps of Kavrepalanchowk and Sindhupalchowk districts with the distribution of outreach centers and health posts are shown in Figs. 2 and 3.

### Participants

#### Eligibility criteria for health centers/health posts

- Outreach centers/health posts located in the Kavrepalanchowk and Sindhupalchowk districts
- Have a nurse (staff nurse or auxiliary nurse midwife (ANM))

#### Eligibility criteria for participants

To resemble the real-world settings as much as possible, we will apply the minimum inclusion and exclusion criteria for the participants. Individual with confirmed pre-existing type II diabetes and prediabetes at the time of our screening (i.e., pre-existing diabetes or prediabetes) or with a confirmed diagnosis of type II diabetes or prediabetes after our community screening process (i.e., newly diagnosed diabetes and prediabetes), who is not planning to relocate outside the current place of living in next 2 years and is older than 18 years of age, will be eligible for project recruitment. People will be excluded if they: are not psychologically capable of communication; and are diagnosed with type 1 diabetes. Participants will be assessed for the risk of diabetes using the Indian Diabetes Risk Score (IDRS) [11]. Anyone identified as at risk will undergo a random finger-prick glucose test followed by the hemoglobin A1c (HbA1c) test. The diagnosis will be confirmed by an HbA1c level ≥ 6.5% for diabetes or between 5.7 and 6.4% for prediabetes [12].

### Screening and recruitment

The program will be rolled out with each outreach center/health post serving as the implementation unit. The program participants will be recruited through a community-based screening effort. Information regarding the screening event will be disseminated at the time of the awareness campaign. Trained diabetes nurses will screen the general population at the outreach center/health post, and those who screen positive will be referred to the DH diabetes clinic for further tests and for program recruitment if eligible for the study. All of the screening procedures including laboratory investigations and anthropometric measurements will be carried out at the respective centers, and all of the longitudinal data of patients will be entered into an electronic health record (EHR) system for data storage and management. Each participant will be assigned a unique identification number at the time of enrollment. All data collected as part of this study will be identified with this number. Research assistants (RAs) will be responsible for obtaining the signed informed consent form from the participants. On the consent form, participants will be asked whether they agree to use of their data should they choose to withdraw from the trial. Participants will also be asked for permission for the research team to share relevant data with people from the universities taking part in the research or from regulatory authorities, where relevant. This trial involves collecting biological specimens for storage.

Recruitment will be primarily through conducting diabetes awareness mass campaigns in the targeted sites of the two districts to achieve adequate participant enrollment. We intend to reach 50,000 people (approximately 2000 people at each of the 9 outreach centers and 17 health posts). Interested candidates will contact the study nurses, who will provide them with basic information about participation, including information on the screening process, time commitment and expectations associated with participation. Assuming 25% of those who are made aware of diabetes will participate in the screening (see later for the details of screening methods), we target screening 12,500 participants. The two-step screening approach will be used for the study after assessing the risk of diabetes among the individuals using the IDRS assessment tool. In the first step, anyone under risk will undergo a random finger-prick blood glucose test using Beurer glucometers [13]. A random blood sugar level of 200 mg/dl or higher will be considered positive for diabetes and a sugar level of 140–199 mg/dl will be considered positive for prediabetes [12]. In the second step, participants screened positive for a random blood glucose level will undergo a glycosylated hemoglobin (HbA1c) test. An HbA1c level between 5.7 and 6.4% will be considered positive for prediabetes and an HbA1c level of 6.5% or above will be considered positive for diabetes [12]. Participants with HbA1c level ≥ 5.7% will be invited to participate in the study. Based on the earlier quoted prevalences of 8.4% (95% CI: 6.2–10.5%) for diabetes [1] and 13% (95% CI: 11.8–14.5%) for prediabetes [14], we expect to identify 1050 (12,500 × 0.084) individuals with diabetes and 1625 (12,500 × 0.13) individuals with prediabetes for this study.

### Randomization

The 26 health clinics will be randomized 1:1 into the continuum of care group (intervention) and the usual care group (control), stratified by outreach center and health post. The usual care group includes those who continue managing their diabetes under the direction of their primary care providers, which is the most commonly used method for diabetes management in Nepal. The selection of the usual care group as a comparator is thus justified. A statistician otherwise not involved in the project will perform this group assignment at the cluster level with simple random selection with the R statistical program. Randomization will be done before participant recruitment. Outcome assessors will be blinded to the group assignment, but the nurses and patient participants will be aware of their group assignment. If blinding to the outcome assessor is accidentally broken, they will follow a standard protocol for reassessment of the patients at another time. Any violations of the study protocol will be recorded and reported to the Ethics Committee.

### Baseline assessment

At baseline, trained RAs will interview the participants using a standardized electronic questionnaire on CommCare, a mobile platform designed for data collection [15]. RAs will receive 2 weeks of training in data collection and ethical issues. The questionnaire will assess socioeconomic characteristics including age, sex, ethnicity, religion, marital status, annual income, education level and family history, and lifestyle factors including smoking, alcohol intake and physical activity. We will use the Global Physical Activity Questionnaire [16] to calculate the metabolic equivalent of task (MET) minutes per week. A weekly MET equivalent of 600 would represent 30 min of brisk walking five times per week or 15 min of running five times per week. We will use PrimeScreen questionnaire [17], a short diet assessment tool to assess the diet quality of the study participants. Body weight will be measured with minimum clothing and without shoes using an Omron Model HBF-400 scale and recorded to the nearest 0.1 pounds. The weighing scale will be calibrated to zero every day. Participants’ heights will be measured, without shoes, while the participants stand against a wall. Height will be measured using a tape measure and recorded to the nearest 0.1 cm.

Blood samples will be collected for HbA1c, low-density lipoprotein (LDL), high-density lipoprotein (HDL), triglycerides and total cholesterol at each respective health center where all the laboratory procedures will be carried out. Blood samples will be collected using evacuated blood collection tubes. Participants will be asked to fast overnight (8–14 h).

### Intervention: NUCOD program components

**Fig. 4:**
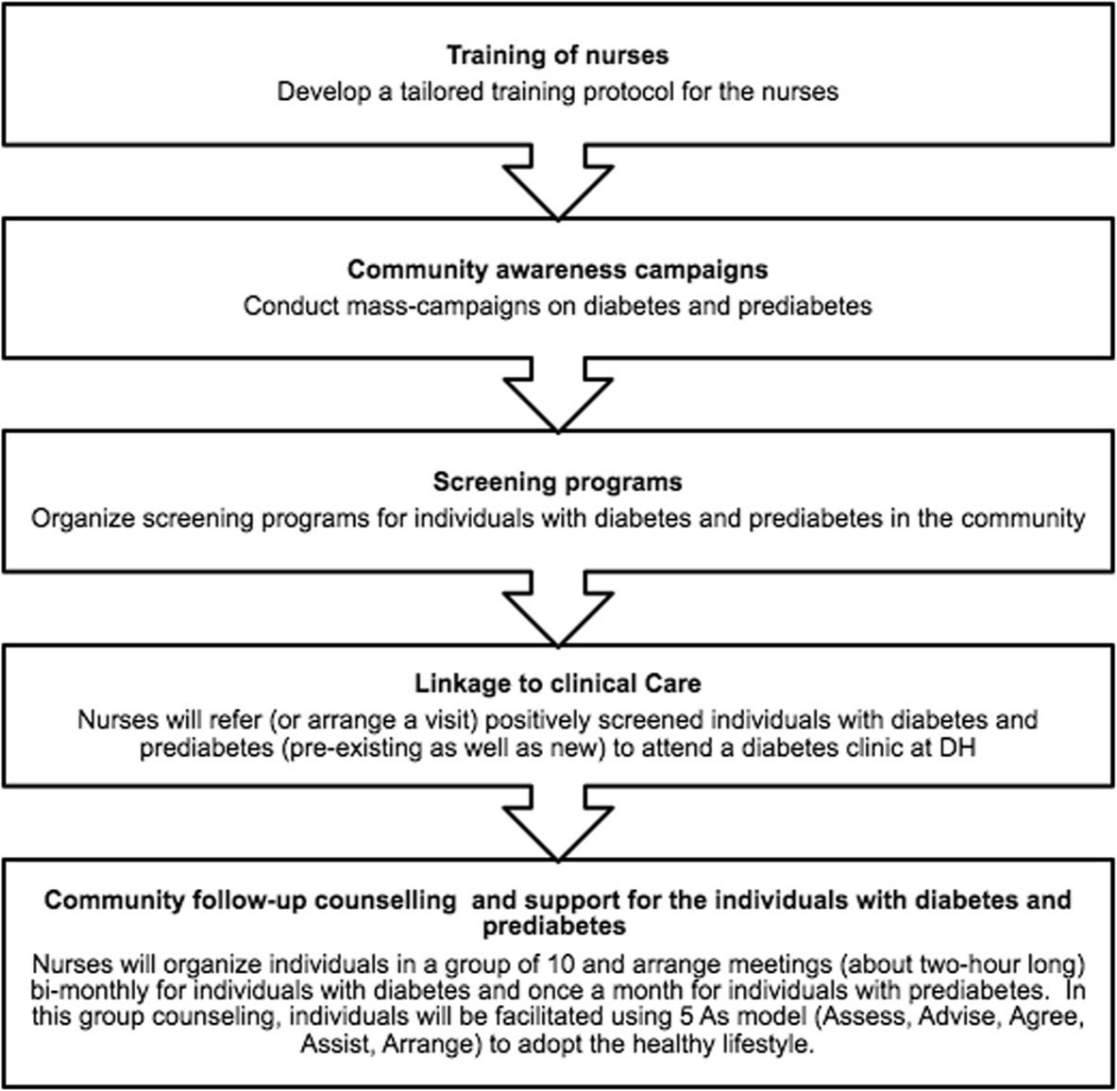
Intervention components of the NUCOD program. DH Dhulikhel Hospital, NUCOD NUrse-led COntinuum of care for people with Diabetes and prediabetes

The intervention will be delivered by specially trained nurses from Dhulikhel Hospital (DH). The intervention has been developed based on our extensive literature review and the recommendations from the Disease Control Priorities-3 (DCP-3) as well as an analysis of local contexts in consultation with various stakeholders. The intervention will include the following components, logically sequenced as a continuum of care approach. Figure 4 presents the major program components. The nurses will act as the leader and coordinator for the implementation of all the program components:

- *▪ Training of nurses*: by drawing from standard training manuals published by the International Diabetes Federation and American Diabetes Association, the project management team will first develop a protocol and content for the training of nurses for this program. This training protocol will be tailored to the context of Nepal and will include case studies and examples relevant to Nepal. The training will focus on clinical as well as case management skills. We will train 26 nurses (one for each site) into diabetes nurses using this training protocol.
- *▪ Community awareness campaigns*: the nurses will lead the organization of a mass campaign on diabetes in coordination with local newspapers, radio stations, youth groups, municipality offices, district health offices and other health facilities. The campaigns will, in particular, include a simple diabetes risk factor assessment tool to be adapted from the Type 2 Diabetes Risk Test (DRT) of the American Diabetes Association [18]. The campaign will encourage people with high risk to attend the screening program.
- *▪ Screening programs*: the nurses will organize screening programs for both diabetes and prediabetes for the community members on specific dates and sites, which will be communicated beforehand to the people in the locality. The screening will be done in coordination with the clinical biochemistry department of DH.
- *▪ Linkage to clinical care*: people who screened positive for diabetes and prediabetes (pre-existing as well as new) will be linked to the DH clinic by nurses, where they will be recruited into the program and will receive their group assignment. People assigned to the control group will follow the usual procedure, while those assigned to the intervention group will attend a 1-h nurse-led counseling session on diabetic care. The patients will be responsible for their medical costs (laboratory investigations and medicine), while people experiencing financial hardship can apply for charity care (free or at a reduced price) with guidance from the nurse. The assigned nurse will be responsible for determining their financial profile by reviewing the annual household income, applicable assets, available insurance coverage and confirmation of other sources of payment.
- *▪ Community follow-up counseling and support for the diabetic patients*: the nurse will organize diabetic patients into groups of 10 and arrange bimonthly meetings (each 2 h in length) in which the patients will be facilitated using the 5A framework (Assess, Advise, Agree, Assist and Arrange follow-up) to adopt a healthy lifestyle (dietary changes, physical activity, cessation of smoking, etc.) [19]. In addition to the lifestyle changes, the participants will also be facilitated to adhere to medical advice (timely follow-up, adherence to medications, etc.). The nurses will also coordinate with the diabetes clinic at the hospital to ensure that the clinical care and the community-level interventions complement each other. They will also have an electronic tablet that has all the details (laboratory results, behavioral parameters, anthropometric measures, etc.) of the participants so that the progress of the participants in these parameters can be recorded and assessed.
- *▪ Prevention programs for prediabetic participants*: in addition to the people with diabetes, the nurses will also form groups of prediabetes participants and will arrange monthly meetings to help facilitate the adoption of a healthy lifestyle using the Diabetes Prevention Program (DPP) curriculum. The DPP lifestyle intervention has been associated with significantly reducing the development of diabetes through its structured behavior-changing approaches [20]. A multidisciplinary team of dieticians and physical therapists will assist with the other components of the DPP program that are related to nutrition and physical activity.

The control group will receive the usual diabetic care, which includes the same hospital quality improvement for diabetic care as already mentioned, the follow-up services from the female community health volunteers (FCHVs) (but without the nurse coordination and supervision as in the intervention group) in addition to the usual care. FCHVs are the grass-roots level of community health workers in Nepal who play important roles in the implementation of community-based programs, including screening for high-risk cases, referring people to local health facilities and maintaining a record of health activities [21]. The program comparison of the intervention group versus the control group is summarized in Table 1.

**Table 1:** Program components for the intervention versus control groups

	Intervention group	Control group
Community awareness campaigns led by trained nurses	x	x
Screening	x	x
Linkage to clinical care	x	
• Coordinated through the nurses	x	
• provided by FCHVs without involvement of the nurse		x
Improved quality of care at the hospital	x	x
Nurse-led counseling	x	
Community follow-up coordinated through the nurses	x	
Prevention program to prediabetics through the nurses	x	

*FCHV* female community health volunteer

### Outcome measures

We will examine both clinical outcomes and implementation outcomes as we hypothesize that the nurse-led continuum of care program will improve both health and the implementation of priority interventions in diabetes. The outcome measure will pertain to the individual participant level. The primary outcome for the diabetes group is the HbA1c level at 12 months from the intervention. The HbA1c level is indicative of the average level of blood glucose over the past 2–3 months [22]. The primary outcome for the prediabetes group is the incidence of diabetes at 12 months from the intervention.

The secondary outcomes are blood pressure, body mass index (BMI) and lipid levels. Also, the secondary outcomes include a series of implementation outcomes in the RE-AIM (reach, effectiveness, adoption, implementation, and maintenance) implementation framework [23–25]. We provide details on those measures in the following (see Table 2 for a summary).

**Table 2:** Measures of outcome variables

	Assessment method	Assessor	Baseline	Midline (6 months)	End line (12 months)	Variable
**Clinical outcomes**						
*Primary outcome*						
Glycemic control (HbA1c)^a^ for diabetes	Blood sample	Biochemist	x	x	x	Continuous
Incidence of diabetes among prediabetes	EHR	RA/nurse		x	x	Binary
*Secondary outcomes*						
Blood pressure	Electronic monitor	RA/nurse	x	X	x	Continuous
Lipid profile	Blood sample	Biochemist	x	X	x	Continuous
Body mass index	EHR^b^	RA/nurse	x	x	x	Continuous
**Implementation outcomes**						
Reach	Log record^c^	RA/nurse		x	x	Continuous
Adoption	SDSCA/DTSQ	RA/nurse	x	x	x	Binary
Implementation (fidelity)	Checklist	SP		x	x	Continuous
Self-monitoring of glucose	Self-reported	RA/nurse		x	x	Binary
Medication adherence	Self-reported	RA/nurse		x	x	Binary

*DTSQ* Diabetes Treatment Satisfaction Questionnaire, *EHR* electronic health record, *HbA1c* hemoglobin A1c (aka glycated hemoglobin), *RA* research assistant, *SDSCA* Summary of Diabetes Self-Care Activities Assessment, *SP* standardized patient

^a^The average level of blood sugar over the past 2–3 months

^b^Calculated as weight in kilograms divided by height in meters squared

^c^Number of people participating in the program divided by the number of people eligible to be recruited into the program

#### Clinical outcomes

The clinicians from Dhulikhel Hospital outreach centers not otherwise associated with the project will measure HbA1c, lipid profiles, BMI and blood pressure when the participants visit the diabetic clinic at baseline, 6 months and 12 months.

HbA1c will be measured using Boronate affinity chromatography (Axis-Shield; Norway) [26], LDL and HDL using the elimination method (Dialab; Austria) [27], triglyceride using GPO-PAP (Dialab; Austria) [28] and total cholesterol using CHOD-PAP (Dialab; Austria) [29]. For each type of assay, the laboratory has quality control (QC) materials (using commercially available assayed and unassayed control material) from Bio-Rad Laboratories, USA. Each QC is run at least in duplicate. External QC is arranged by internationally recognized reference laboratories that distribute batches of samples of various concentrations for each assay. The laboratory performs the External Quality Assurance Scheme from an unknown assayed sample from the Department of Clinical Biochemistry CMC, Vellore, India for 23 routine parameters, 5 immunological parameters and HbA1c. Additionally, 5% of the blood samples will be obtained in duplicate and sent for testing of all parameters, blinded to the laboratory personnel.

The mean of three measurements of systolic and diastolic blood pressure, using a Microlife automatic blood pressure measuring device, will be adopted for analysis. Hypertension is defined as systolic blood pressure ≥ 140 mmHg or diastolic blood pressure ≥ 90 mmHg or taking antihypertensive medication [30]. Weight will be measured and recorded to the nearest 0.1 kg, without shoes and with minimum clothing, using an Omron Model HBF-400 scale. Height will be measured without shoes using a standard tape measure with participants standing against a wall for measurement, and recorded to the nearest 0.1 cm. The BMI will be calculated as weight in kilograms divided by height in meters squared. Overweight is defined as a BMI of 25 kg/m^2^ or higher and obesity is defined as BMI of 30 kg/m^2^ or higher based on international cutoff points [31].

#### Implementation outcomes

The RE-AIM (Reach, Effectiveness, Adoption, Implementation, and Maintenance) framework will be implemented as follows:

- *Reach* will be measured by the number of people participating in the program divided by the number of people eligible to be recruited into the program.
- *Effectiveness* will be represented by the clinical outcomes.
- *Adoption* at the patient level will be measured by the proportion of people adherent to the clinical advice in lifestyle and self-care—this will be measured by the self-reported Summary of Diabetes Self-Care Activities (SDSCA) scale [32] at baseline, 6 months and 12 months (the SDSCA measure is a brief self-report questionnaire that includes items assessing general diet, specific diet, exercise, blood-glucose testing, foot care and smoking) and, in addition, the Diabetes Treatment Satisfaction Questionnaire (DTSQ) [33] will be administered at baseline, 6 months and 12 months (the DTSQ is the most commonly used patient-reported outcome in diabetes trials, which reflects the patient’s perception of the treatment)—and at the clinic level will be measured by the proportion of health clinics successfully recruited into the program over the clinics eligible and approached to participate in the program.
- *Implementation* will examine the fidelity and quality of the program execution per protocol. To check program fidelity, we will select and train standardized patients (SPs) from the program participants with stable conditions—these SPs will serve as the “secret agents” and the sentry to assess program fidelity with a quality checklist through their routine encounters with the clinicians at the DH diabetes clinic and the nurses; the clinicians will be blinded to the status of the SPs; and the development of SPs and the checklist will follow the protocol we have developed in a separate study [34].
- *Maintenance* will not be assessed for the purpose of this study.

The hospital and program administrative system will collect a range of other information including detailed program costs, health service utilization and incidence of complications and comorbidities. All data will be entered and securely stored in the EHR, a secured online data capturing and management system developed for the study at the DH.

### Sample size

In this proposed community-based intervention, changes in the HbA1c levels (continuous outcome) were considered for calculating the sample size for the individuals with diabetes and the incidence of diabetes (binary outcome) was considered for calculating the sample size for the individuals with prediabetes, respectively. For the individuals with diabetes, we aim to detect a clinically significant reduction in HbA1c levels from 7.6% to 6.5% (SD = 1.5%) (based on our assumption, the recommended effective diabetic management level and results of previous studies) among the individuals with diabetes during the 12-month period [35, 36] with medium effect size [37]. We expect a reduction of HbA1c to 7.0% in the control group, an optimal target level defined by the American Diabetes Association (ADA) [38]. Setting the statistical significance at the 0.05 level, seeking 90% power, with an intraclass correlation coefficient (ICC) of 0.01 based on a previous study conducted in Nepal [39], an average cluster size of 50 (individuals with diabetes) based on preliminary data from the awareness and screening campaign, a design effect for clustering of 1.49 and a design effect for unequal cluster size of 1.52 (coefficient of variation of cluster size 0.25), 91 people with diabetes per arm (182 total) will be required for the study. For the incidence of diabetes among people with prediabetes (dichotomous outcome), we considered the incidence of 10% and 2%, respectively, for the two groups; stated as a range in an earlier study [40]. Based on these considerations, 471 people with prediabetes per arm (942 total) will be required. We expect a dropout rate of 10% from baseline to 12-month follow-up; hence, we will need to recruit a total of 200 diabetes and 1036 prediabetes participants (total sample size = 1236 participants). The sample size for our cluster RCT was calculated using the following equation. Details of the sample size estimation is available in another study conducted by Ribeiro et al. [41]:

$$ {\mathrm{SS}}_{\mathrm{cluster}\ \mathrm{RCT}}={\mathrm{SS}}_{\mathrm{standard}\ \mathrm{RCT}}\times {\mathrm{DE}}_{\mathrm{cluster}}\times {\mathrm{DE}}_{\mathrm{unequal}} $$

where SS_cluster RCT_ = total sample size for a cluster RCT; SS_standard RCT_ = total sample size for a standard RCT (equal to (Z_1 – α / 2_ + Z_1 − β_)^2^ 2σ^2^ / Δ^2^, where *Z*_*x*_ is the *x*th percentage point of the standard normal distribution, Δ is the clinically important difference in treatment means and σ^2^ is the variance in the outcome); DE_cluster_ = design effect for clustering (equal to 1 + ICC × (m – 1), where *m* is the number per site); and DE_unequal_ = design effect for unequal cluster size (equal to 1 + [(1 + *cv*^2^) × *m* − 1] × ICC, where *cv* is the coefficient of variation of cluster size and *m* is the mean cluster size).

### Statistical analysis

Data will be analyzed at the individual (patient) level. Statistical analyses will be performed using the intention-to-treat (ITT) approach in the originally assigned groups. Demographic and baseline characteristics for the intervention and control groups will be presented in the form of mean (standard deviation (SD)) or 95% confidence intervals (CIs) for continuous variables and counts (percentages) for categorical variables. ITT analysis will be performed on the final data collected at 12 months.

Generalized estimating equations (GEEs) with clustering by site, an exchangeable correlation matrix and robust variances will be used to assess the program effect adjusting for potential baseline covariates. The model will take account of clustering and will be used to look at the difference between groups for the HbA1c level at 6 and 12 months, respectively. Multiple imputation will be used to account for the missing values assuming they are missing at random. To test for the secondary sensitivity analysis, data will be analyzed without multiple imputation and without baseline covariate adjustment. The difference in the incidence of diabetes between the groups among the individuals with prediabetes will be tested using a GEE. Statistical significance will be assessed at the 5% level and all analyses will be two-sided. All data analyses will be performed using the Stata 15 (StataCorp, TX, USA) statistical software program.

As we expect that the program effect may differ between several subgroups of the program participants, we plan to do secondary analyses of the following three subgroups for the primary (HbA1C) and secondary (incidence of diabetes) outcomes: the subgroups with poor initial glycemic level (HbA1c ≥ 5.7%) (people with poorer glycemic level at baseline may have a stronger desire to change their behavior); the subgroups divided by gender (in Nepal, women tend to be more adherent to clinician guidance); the subgroups of different social economic classes (people in higher socioeconomic class may be more likely to adhere to lifestyle changes); and government versus nongovernment health centers. The secondary analyses will compare the HbA1c level and incidence of diabetes among people with prediabetes among these subgroups at the end of the 12-month intervention using the generalized linear mixed models with a random cluster effect and adjusting for potential confounders.

Descriptive statistics (i.e., counts, means and proportions) will be calculated to evaluate the reach, effectiveness, adoption and implementation of the program.

#### Economic analysis

Cost-effectiveness analysis will also be conducted with a Markov simulation model to be developed. The Markov model has been widely used to describe the development of the chronic noncommunicable diseases [42]. All direct costs (e.g., screening cost, cost of training of nurses and cost of EHR system development) and indirect costs will be collected. All costs will be reported in 2020 US dollars using the exchange rate of 1 USD = 111.7 NPR. We will solve the model numerically over a short and long timeframe (i.e., 10 years and 20 years), and calculate the incidence of diabetes, costs and quality-adjusted-life-years (QALYs) under the control case or the NUCOD program. Incremental QALY and costs of the NUCOD program relative to the control case will be calculated with an annual discount rate of 3%. We will use WHO-CHOICE (Choosing Interventions that are Cost-Effective) thresholds for cost-effectiveness: an intervention is defined as “cost-effective” if it produces a healthy life year for less than three times the gross domestic product (GDP) per capita, and as “very cost-effective” if it produces a healthy life year for less than the GDP per capita [43, 44].

### Data monitoring and quality assurance

The trial will receive overall supervision from a Trial Steering Committee (TSC) who will conduct quarterly on-site monitoring of health clinics (with repeat visits to sites where performance is a concern), monitor trial progress and review the quality and completeness of data. The TSC will include an independent chairperson not involved directly with the study, one clinical and one methodological expert who are also not involved with the study, and a principal investigator of the study. Our trained diabetes nurses will be responsible for all aspects of local organization, such as identifying and recruiting participants, and RAs will be responsible for obtaining informed consent. A formal data monitoring committee (DMC) was not considered for the conduct of this study as this will be a low-risk intervention; however, the trial will be reviewed by national (every 6-month) and institutional (every 2-month) Ethics Committee Review Board members throughout the trial period. A Stakeholder and Public Involvement Group (SPIG) was also not formed as the study will be mainly led by the trained diabetes nurses.

## Discussion

To our knowledge, this study is the first to include a sizable number of diabetes and prediabetes participants in a community-based setting and a continuum of care program run by diabetes nurses. The priority health problems of the target population, the potential solution of the problem, recruitment strategies and intervention delivery formats were discussed early on by the study-specific collaborative partners in DH and the community, which will lead to the success of the program.

The results of this study may provide much-needed evidence for the policy-makers on the clinical effectiveness, cost-effectiveness and implementation effectiveness of nurse-led continuum of care for people with diabetes and prediabetes in resource-limited settings. Many low-income countries now face a double burden of chronic diseases and infectious diseases. But large trials have been rarely conducted in those settings in low-income countries. This proposal has several unique features. First, although nurse-led diabetes prevention and management programs have been tested earlier in different settings, this is unique because it addresses the full continuum of care from the level of awareness and detection to management and follow-up all led by nurses. Second, the study focuses not only on clinical outcomes but also on implementation outcomes. The nurse-led continuum of care may theoretically improve health among people with diabetes but also improve adoption of DCT-3-recommended and evidence-based individual intervention elements. We will also use some innovative strategies to assess program implementation fidelity and quality, such as the use of standardized patients. Third, the study builds a culturally tailored and locally contextual training program for nurses utilizing the existing training programs of the International Diabetes Federation and the American Diabetes Association. Fourth, the study utilizes a unique setting of suburban tertiary-level community-based health institutions that have an extensive network of health centers in rural areas as well. Hence, the experience we gain from this project will be easily translated into larger health care systems. Fifth, we have designed the trial, taking implementation, feasibility and sustainability into full consideration. For instance, we did not offer free care to all program participants in the trial as that will not be sustainable once the program stops.

Although this trial may hold promise for better clinical and implementation outcomes, it is not without potential limitations. We will have a large sample size, but the setting of the study may not represent the entire nation, thus limiting the generalizability of our findings. However, our setting will cover both public and private health sectors and may serve as a proxy for the Nepalese population.

## Trial status

The protocol is the first version as we have not changed any information after registering in the ClinicalTrials.gov registry on 18 October 2019. We have not yet started the recruitment of our sample. The approximate date when recruitment will be completed will be the end of December 2020.

## Data Availability

The proposed project is a collaborative effort between investigators at SYSU and KU. The aforementioned institutions will jointly share ownership of the data. Study investigators at SYSU and KU will have access to the data. Data can be obtained from the corresponding author upon reasonable request.

## Abbreviations

ADA: American Diabetes Association
BMI: Body mass index
DCP-3: Disease Control Priority-3
DH: Dhulikhel Hospital
DPP: Diabetes Prevention Program
DRT: Diabetes Risk Test
EHR: Electronic health record
FCHV: Female community health volunteer
HDL: High-density lipoprotein
ICC: Intraclass correlation coefficient
IDRS: Indian Diabetes Risk Score
ITT: Intention to treat
LDL: Low-density lipoprotein
MET: Metabolic equivalent of task
NPR: Nepalese Rupee
QALY: Quality-adjusted life years
QC: Quality control
USD: US Dollar
